# Working Memory, Attention Control, and Vocabulary Retention in AI (ChatGPT)-Assisted Foreign Language Learning: A Structural Cognitive Modelling Approach

**DOI:** 10.3390/jintelligence14040062

**Published:** 2026-04-08

**Authors:** Mohammad Hamad Al-khresheh, Mayez Almayez, Shatha F. Alruwaili

**Affiliations:** 1 Humanities and Social Research Center, Northern Border University, Arar 73213, Saudi Arabia; 2 English Language Department, College of Arts, University of Ha’il, Ha’il 55473, Saudi Arabia; 3 Department of English Language and Literature, College of Languages and Translation, Imam Mohammad Ibn Saud Islamic University (IMSIU), Riyadh 11432, Saudi Arabia

**Keywords:** attention control, ChatGPT-assisted learning, cognitive regulation, foreign language learning, vocabulary retention, working memory

## Abstract

This study examined how working memory, attention control, and frequency of ChatGPT-4 use are structurally associated with vocabulary retention in foreign language learning. A quantitative cross-sectional survey design was employed, with data collected from 1002 EFL learners via stratified random sampling. Validated self-report instruments measured working memory, attention control, frequency of ChatGPT use, and vocabulary retention (immediate recall, delayed retention, semantic integration, and productive use). Structural equation modelling was used to test the proposed model. The results showed that working memory was strongly associated with attention control and exerted a direct effect on vocabulary retention across all dimensions. Attention control explained a substantial share of the relationship between working memory and retention, indicating that regulatory allocation of attention, rather than memory capacity alone, governs whether lexical information is stabilised during ChatGPT-assisted learning. The frequency of ChatGPT use conditioned these cognitive pathways by strengthening links between working memory and attention control, and between attention control and vocabulary retention, at higher levels of engagement. Frequency did not predict retention independently, indicating that repeated use supports learning only to the extent that it reinforces cognitive regulation rather than increasing exposure. Vocabulary learning with AI relies more on cognitive regulation and engagement than exposure.

## 1. Introduction

Digital mediation has become an established feature of foreign language learning, altering the conditions under which learners encounter and practise new vocabulary (Bahari et al., 2023). Recent developments in conversational language systems have intensified this shift by introducing environments where learners engage with extended, responsive linguistic input while simultaneously producing language (Orak, 2025; Weerasinghe et al., 2022). Such settings differ fundamentally from earlier digital tools that delivered fixed content or discrete feedback (R. Feng et al., 2023; Yusuf, 2025). Vocabulary learning within conversational systems unfolds through continuous interaction, requiring learners to manage incoming information, maintain focus on task goals, and regulate competing lexical alternatives during use.

Vocabulary acquisition is widely recognised as cognitively demanding, particularly in foreign language contexts where learners must retain unfamiliar forms, map meanings across contexts, and retrieve items accurately during production (Ellis, 2019). These demands become more pronounced when learning takes place through conversational interfaces. Learners interacting with ChatGPT are exposed to dense lexical input, multiple reformulations, and immediate prompts for response, a configuration known to increase processing load and attentional demands in interactive settings (Qiu et al., 2025). Each interaction requires decisions about what to attend to, what to ignore, and how to integrate new lexical material into ongoing discourse, processes closely associated with core cognitive resources rather than exposure alone (Czerwińska & Piskorska, 2018).

Despite the rapid adoption of generative conversational tools in language learning, research has largely approached these environments from descriptive or evaluative perspectives. Much of the existing work concentrates on learners’ perceptions, reported usefulness, or short-term task outcomes, particularly in studies examining engagement with systems such as ChatGPT (Xu et al., 2024; Zaim et al., 2025). While informative, this line of inquiry offers limited insight into the cognitive mechanisms governing vocabulary learning in interactive settings. In particular, the role of working memory in managing lexical information during interaction, and the function of attention control in regulating focus and selection, remain insufficiently theorised and empirically specified within conversational learning contexts (L. Feng, 2024). Existing studies rarely examine how these cognitive resources jointly regulate the processing and retention of lexical input during conversational AI interaction, leaving an important gap in current accounts of AI-assisted vocabulary learning.

Another limitation of current research lies in the treatment of vocabulary retention as a single outcome. Vocabulary knowledge develops across multiple dimensions, ranging from immediate recall to longer-term retention, semantic integration, and productive use, as consistently demonstrated in empirical vocabulary research (Teng, 2022; Webb et al., 2023). These dimensions place different demands on learners’ cognitive resources and are unlikely to be supported by identical mechanisms. Without distinguishing among these outcomes, it is difficult to explain variability in learning trajectories or to account for why some learners benefit more from conversational systems than others under comparable conditions (Teng & Zhang, 2023).

Addressing these issues requires a shift from tool-centred evaluation to cognitively grounded explanation. Working memory and attention control provide a principled basis for examining how learners process, regulate, and retain lexical information during conversationally mediated learning, given their established role in managing interference, maintaining task goals, and supporting controlled retrieval (Bialystok & Craik, 2022; Friedman & Robbins, 2021). The present study adopts this perspective by focusing on these two regulatory mechanisms, which govern how learners maintain lexical information, allocate attention to relevant input, and coordinate comprehension with production during interaction with conversational AI systems. Examining these constructs within a structural modelling framework enables explicit testing of direct relationships, indirect pathways, and contextual conditions that shape learning outcomes.

The present study develops a structural cognitive model to examine how working memory and attention control relate to vocabulary retention in ChatGPT-assisted foreign language learning. Vocabulary retention is conceptualised as a multidimensional outcome encompassing immediate recall, delayed retention, semantic integration, and productive use, consistent with contemporary vocabulary research. In addition, the study examines how the frequency of ChatGPT use shapes the relationship between cognitive resources and learning outcomes. Within this framework, the hypotheses derive from cognitive accounts of language processing, which posit that lexical learning depends on the regulation of attention and the maintenance and updating of linguistic representations during interaction. The central objective is to explain how cognitive processes operate within conversational learning environments, thereby advancing a theoretically grounded account of vocabulary learning appropriate for contemporary foreign language contexts.

## 2. Literature Review

### 2.1. Working Memory and Attention Control in Foreign Language Learning

Foreign language learning places learners under persistent cognitive pressure, particularly when lexical processing occurs in contexts characterised by competition, uncertainty, and limited processing capacity. Research in cognitive psychology frames these conditions as situations in which domain-general control systems are recruited to manage selection and interference during language use (Bialystok & Craik, 2022; Friedman & Robbins, 2021). In second- and foreign-language contexts, lexical access commonly involves the simultaneous activation of multiple word candidates, sometimes across languages, requiring mechanisms that regulate attention and suppress competing representations (Luque & Morgan-Short, 2021; Zhu et al., 2021).

Attentional control has been shown to play a central role in resolving such competition. Luque and Morgan-Short (2021) report that individual differences in reactive control are associated with second-language proficiency, particularly on tasks involving interference resolution. Neuroimaging evidence further indicates increased engagement of control-related regions when learners must suppress competing lexical alternatives under forced or externally constrained language selection (Zhu et al., 2021). At the behavioural level, research on Stroop interference demonstrates that attentional selection operates across multiple processing stages, supporting the suppression of task-irrelevant verbal information during language tasks (Parris et al., 2021). Complementing these findings, network-level analyses distinguish regions responsible for linguistic representation from those involved in regulating access to such representations, with control regions overlapping domain-general systems associated with selection and inhibition (Fedorenko & Shain, 2021; Hodgson et al., 2021).

Working memory contributes to language learning by supporting the temporary maintenance and manipulation of linguistic information under distraction (Li, 2023). Studies of vocabulary learning under multimedia conditions show that executive working memory and phonological short-term memory predict both immediate and delayed vocabulary outcomes, particularly when learners must integrate multiple sources of input or manage competing information streams (Teng & Zhang, 2021). From a theoretical perspective, working memory capacity is increasingly understood to reflect the ability to sustain attention on task-relevant representations in the presence of interference, rather than as a passive storage system (Bialystok & Craik, 2022; Friedman & Robbins, 2021). Evidence from large-scale language processing research further indicates that lexical processing difficulty reflects uncertainty among competing candidates and depends on the maintenance and updating of contextual information in working memory (Fedorenko & Shain, 2021; Friedman & Robbins, 2021; Shain et al., 2024).

Recent accounts of bilingual cognition interpret differences in executive functioning through the lens of attentional control efficiency. Evidence indicates that experienced bilinguals regulate attention more economically, achieving comparable or superior performance with reduced engagement of control-related neural resources (Bialystok & Craik, 2022). Findings from conflict-monitoring and task-switching paradigms attribute this pattern to more effective allocation of attentional resources rather than stronger inhibitory mechanisms alone (Friedman & Robbins, 2021). These results align with models that position attention control as the mechanism regulating competing representations during language use (Luque & Morgan-Short, 2021). The interaction between working memory and attention control becomes particularly visible in complex language tasks such as writing. Research on second-language writing shows that working memory is weakly associated with overall proficiency yet reliably predicts performance dimensions such as accuracy, complexity, and fluency when task demands increase (Kormos, 2023; Li, 2023). Evidence further indicates stronger effects of working memory capacity in cognitively demanding genres, where limitations in attentional resource allocation constrain performance under higher reasoning load (J. Zhang & Zhang, 2023). Across studies, different components of working memory support distinct stages of the writing process, suggesting that successful performance depends on coordinated regulation rather than a single capacity constraint (Bourzeg, 2025; Kormos, 2023; Li, 2023).

Language-based self-regulation provides an additional link between working memory, attention control, and learning behaviour. Nedergaard et al. (2022) show that inner speech supports attention direction, goal maintenance, and inhibition during tasks requiring memory and categorisation, with verbal interference selectively disrupting performance when covert linguistic rehearsal is required. These findings suggest that language-mediated self-cueing is part of the control architecture that supports task regulation. In second-language contexts, such self-regulatory processes depend on learners’ ability to maintain linguistic representations and to control attention in the face of competing stimuli (Bialystok & Craik, 2022; Friedman & Robbins, 2021; Kormos, 2023; Li, 2023; Nedergaard et al., 2022).

Research on implicit learning qualifies accounts that prioritise sustained top-down control by showing that reduced executive engagement can, under specific conditions, facilitate automatic pattern extraction. Smalle et al. (2022) report that temporary depletion of prefrontal control enhances implicit statistical learning, including phonotactic sensitivity and word segmentation, indicating that strong attentional regulation may inhibit certain forms of automatic learning. This finding aligns with broader cognitive accounts suggesting that executive resources support explicit, goal-directed processing while constraining incidental learning mechanisms when control demands remain high (Bialystok & Craik, 2022; Friedman & Robbins, 2021).

In foreign language learning, these observations situate working memory and attention control within a broader system of cognitive regulation that governs how lexical information is maintained, selected, and integrated during language processing. Research in bilingual language processing indicates that lexical access frequently involves competition among alternative representations, requiring mechanisms that sustain task-relevant information while suppressing interference from competing candidates (Luque & Morgan-Short, 2021; Zhu et al., 2021). Cognitive accounts further interpret working memory as a system that maintains and updates linguistic representations under conditions of interference rather than as a passive storage mechanism (Bialystok & Craik, 2022). In interactive learning environments, such regulatory processes become particularly consequential because learners must coordinate comprehension, monitoring, and production while evaluating multiple lexical alternatives during ongoing discourse. Attention control, therefore, regulates the allocation of processing resources toward learning-relevant input and the suppression of distracting alternatives, particularly when linguistic input is dense and rapidly evolving (Friedman & Robbins, 2021). This perspective situates vocabulary learning in conversational environments as a cognitively regulated process shaped by the coordination of executive resources rather than by exposure alone.

### 2.2. Vocabulary Retention as a Multidimensional Cognitive Outcome

Vocabulary retention in second- and foreign-language learning is widely conceptualised as a multidimensional construct rather than a single outcome. Meta-analytic and large-scale reviews show that vocabulary knowledge varies across time, representational depth, and mode of access, with each dimension reflecting distinct memory processes rather than alternative measurement practices (Hao et al., 2021; Webb et al., 2023). This perspective has led to a shift away from global vocabulary scores toward analytically differentiated outcomes in empirical research (Calvo-Ferrer & Belda-Medina, 2021).

A central analytical distinction concerns the temporal dimension of vocabulary retention, since immediate recall and delayed retention represent different memory states rather than interchangeable indicators of learning. Immediate recall reflects short-term encoding processes, whereas delayed retention reflects post-encoding consolidation and resistance to interference (Teng, 2022). Intervention studies show that delayed performance typically remains lower than immediate recall while remaining above baseline levels, indicating partial consolidation rather than simple decay (Ahmed et al., 2022; Zakian et al., 2022). Retention stability depends on encoding conditions and subsequent practice. Research in immersive and multimedia environments shows that distinctive contextual encoding reduces retrieval interference and supports delayed retention through stronger context–item associations (Essoe et al., 2022; Weerasinghe et al., 2022). Evidence on spaced retrieval further indicates that repeated exposure reactivates lexical representations over time and moderates forgetting trajectories without eliminating decline entirely (R. Feng et al., 2023; Kazu & Kuvvetli, 2023). Retention outcomes also differ according to the mode of lexical access. Meta-analytic evidence indicates that receptive knowledge is typically acquired and maintained more consistently than productive knowledge, particularly under delayed testing conditions, in which retrieval demands increase (Hao et al., 2021; Webb et al., 2023). Productive retention requires controlled retrieval, precise coordination between form and meaning, and sustained attentional regulation during use, conditions that place heavier demands on cognitive control processes (Teng & Zhang, 2021).

Productive vocabulary retention is closely associated with learning conditions that require repeated retrieval and generative use rather than recognition-based processing. Empirical evidence from interaction-oriented tasks shows that game-based learning and chatbot-mediated activities support stronger productive gains when learners actively deploy newly acquired lexical items in meaningful contexts (Kazu & Kuvvetli, 2023; Z. Zhang & Huang, 2024). Spoken-output tasks further connect productive retention to phonological rehearsal and articulatory planning, with automatic speech recognition environments strengthening phonological representations through repeated production cycles (Bashori et al., 2021, 2022). Beyond temporal stability and access mode, vocabulary retention also varies along a dimension of representational depth. Research on multimedia, augmented reality, and virtual reality learning environments frames deeper retention as semantic integration, where lexical items are embedded within context-rich representations rather than maintained as isolated form–meaning associations (Teng, 2022; Yilmaz et al., 2022). Comparative studies consistently report superior immediate and delayed retention under such conditions relative to decontextualised learning formats, indicating that representational depth contributes independently to retention durability (Weerasinghe et al., 2022; Zakian et al., 2022).

Semantic integration is further supported by learning conditions that promote personal relevance and conceptual organisation. Learner-generated content has been shown to produce stronger retention than externally selected items, indicating the role of meaningful engagement in structuring lexical knowledge (Lambert et al., 2021). Similarly, mind-mapping techniques and communicative game contexts support retention by organising vocabulary around conceptual relationships rather than isolated forms (Calvo-Ferrer & Belda-Medina, 2021; R. Feng et al., 2023).

Vocabulary retention is closely associated with underlying cognitive processes. Attention and noticing function as prerequisites for encoding, with interactive and multimodal tasks increasing attentional engagement during learning (Weerasinghe et al., 2022; Yilmaz et al., 2022). Working memory supports the maintenance and manipulation of novel lexical forms and meanings during encoding and retrieval, with executive capacity predicting receptive and productive gains across multimedia learning conditions (Hao et al., 2021; Teng & Zhang, 2021). From this perspective, vocabulary retention is a structured cognitive outcome comprising temporally distinct and representationally differentiated components. Immediate recall, delayed retention, receptive knowledge, productive use, and semantic integration reflect related yet non-equivalent memory processes shaped by attention allocation, working memory capacity, consolidation dynamics, and retrieval demands (Calvo-Ferrer & Belda-Medina, 2021; Teng, 2022; Webb et al., 2023).

### 2.3. Cognitive Processing in ChatGPT-Assisted Foreign Language Learning

Conversational AI interaction places sustained pressure on attentional regulation because learners must coordinate comprehension, response formulation, and monitoring of form–meaning relations within a single activity stream. Czerwińska and Piskorska (2018) show that foreign language processing relies on attentional resources distributed across perception, memory, and planning, with interference increasing as task demands accumulate. Neurocognitive evidence from immersive and interactive second-language contexts further indicates that such conditions recruit executive-control networks and increase processing effort during early engagement. However, prolonged exposure may be associated with more efficient allocation of attentional resources (Gkintoni et al., 2025). Task structure further constrains this process. Evidence from synchronous computer-mediated oral interaction indicates that excessive task complexity can reduce effective engagement, suggesting that conversational interaction alone does not guarantee sustained attentional focus (Qiu et al., 2025). Within AI-mediated interaction, attention regulation therefore operates as a limiting condition that determines whether engagement supports structured learning or fragmented processing.

Generative conversational environments expose learners to dense lexical input that requires continuous prioritisation rather than passive reception. From a usage-based perspective, Ellis (2019) frames language learning as sensitivity to distributional regularities under attentional constraint, placing selective attention at the centre of lexical development. Empirical task-based research supports this position. Teng and Zhang (2021) report that vocabulary gains are closely associated with learners’ ability to plan, monitor, and evaluate lexical processing even under high involvement load. When multimedia input is paired with sentence production, vocabulary outcomes improve while cognitive demands increase substantially, making regulation a decisive factor in learning outcomes (Teng & Zhang, 2023). Task design further shapes lexical focus: divergent communicative tasks elicit proportionally greater attention to lexical items than convergent tasks (Kim & Kerr, 2024), whereas research on spoken interaction shows that learners manage lexical difficulty through compensatory strategies when retrieval becomes demanding (Chou, 2024). These patterns indicate that lexical regulation, rather than input availability, constrains vocabulary development in conversational AI settings.

From a cognitive load perspective, generative systems can either stabilise or destabilise learning depending on how system features align with learner capacity. Reviews of computer-assisted language learning, framed within cognitive load theory, demonstrate that instructional features reduce unnecessary processing only when they align with learners’ proficiency levels and task demands (Bahari et al., 2023). Evidence from AI-assisted language learning further suggests that adaptive feedback and pacing can reduce perceived processing burden while supporting performance; L. Feng (2024) attributes this effect to responsiveness rather than automation. Expert-based analyses focusing on conversational AI describe a tension between support and overload. Xu et al. (2024) document how curated input may assist regulation, while information abundance places pressure on attentional control. Classroom-based evidence reinforces this concern, with learners reporting manageable effort and perceived usefulness, alongside recognition of dependency and of risks of surface-level engagement (Zaim et al., 2025). Within lexical pedagogy, Tangpijaikul (2025) argues that AI-driven feedback is most effective during output generation, where processing demands peak, and targeted feedback can direct attention without removing learner responsibility.

Task-based research shows that cognitive demands shape performance through trade-offs rather than uniform improvement. Experimental evidence indicates that increased task complexity can support lexical diversity and accuracy when planning resources are available, while fluency deteriorates once attentional limits are exceeded (Santos, 2018). Affective research aligns with this pattern. Chen (2023) reports that lexical retrieval difficulties and idea-generation problems drive moment-to-moment declines in enjoyment during interactive speaking tasks, linking cognitive load directly to disengagement. Embodied learning studies further complicate this relationship. Schmidt et al. (2019) show that added motor demands increase perceived effort while supporting retention, indicating redistribution rather than accumulation of cognitive demands. These findings caution against equating richer interaction with improved learning and point to the need for careful calibration of task demands in conversational AI contexts.

Learning in conversational AI environments can be understood as regulated participation in high-density informational settings rather than effortless acquisition. Language cognition research identifies attention as the mechanism determining which input becomes learning-relevant; Ellis (2019) characterises attention as a gate on acquisition. Nussbaum and Bekerman (2025) similarly describe generative systems as cognitive co-participants that redistribute planning and monitoring between learner and system, supporting performance under load while risking reduced self-regulatory control when automation dominates. Evidence reviewed here indicates that learning outcomes in AI-mediated foreign language contexts depend largely on learners’ ability to regulate attention, manage lexical density, and maintain control over form–meaning decisions.

### 2.4. Research Gap and Hypothesis Development

Research on foreign language vocabulary learning has consistently shown that working memory and attention control are central to managing lexical information under conditions of competition, uncertainty, and limited processing capacity, as demonstrated across cognitive and second language processing studies examining the regulation of lexical information under constrained processing conditions (Bialystok & Craik, 2022; Friedman & Robbins, 2021; Luque & Morgan-Short, 2021; Zhu et al., 2021). Evidence from task-based interaction, multimedia learning, and technology-mediated instruction indicates that vocabulary outcomes depend on learners’ capacity to maintain task-relevant representations, regulate attention, and suppress competing lexical alternatives, particularly in environments characterised by dense input and concurrent processing demands (Hao et al., 2021; Teng & Zhang, 2021, 2023; Webb et al., 2023). At the same time, vocabulary retention has been shown to consist of multiple non-equivalent outcomes, including immediate recall, delayed retention, semantic integration, and productive use, each associated with distinct cognitive demands rather than a single underlying mechanism (Calvo-Ferrer & Belda-Medina, 2021; Teng, 2022; Webb et al., 2023; Zakian et al., 2022). Despite this evidence, existing studies tend to examine working memory (e.g., Hao et al., 2021; Li, 2023) or attention control (e.g., Kormos, 2023; J. Zhang & Zhang, 2023) in isolation and frequently operationalise vocabulary retention as a unitary construct, rather than modelling how these cognitive mechanisms jointly contribute to differentiated retention outcomes, thereby limiting explanatory precision regarding their combined effects.

In the present study, working memory and attention control are examined jointly because these regulatory mechanisms govern how learners maintain lexical representations, allocate attention to relevant linguistic input, and manage competing alternatives during interactive language use. In conversational AI environments, these processes become particularly salient as learners must interpret dense input while simultaneously generating responses. Examining these mechanisms together, consequently, provides a theoretically grounded basis for explaining variability in vocabulary retention across different outcome dimensions.

Empirical work on systems such as ChatGPT has primarily focused on learner perceptions, reported usefulness, or aggregate performance indicators (e.g., AlTwijri & Alghizzi, 2024; Xu et al., 2024; Zaim et al., 2025; Z. Zhang & Huang, 2024), with limited attention to the cognitive mechanisms governing vocabulary learning during interaction. Although interactive AI environments impose sustained demands on attentional regulation and lexical processing, existing studies (e.g., Bahari et al., 2023; L. Feng, 2024; Qiu et al., 2025; Tangpijaikul, 2025) have not explicitly modelled how working memory and attention jointly shape vocabulary retention across differentiated outcome dimensions. In addition, frequency of engagement with conversational AI is typically treated as a descriptive usage variable (e.g., Qiu et al., 2025; Xu et al., 2024; Zaim et al., 2025), rather than as a conditioning factor that may alter the strength of cognitive relationships during learning.

These gaps point to the need for a structurally specified, cognitively grounded model capable of testing direct relationships, indirect pathways, and conditional effects among working memory, attention control, and differentiated vocabulary outcomes in ChatGPT-assisted foreign language learning. Therefore, the study seeks to answer this main research question:

- To what extent do working memory and attention control predict vocabulary retention in ChatGPT-assisted foreign language learning, and how are these relationships mediated by attention control and moderated by frequency of ChatGPT use?

To address this question, the following hypotheses are formulated based on the reviewed literature and the cognitive framework outlined above, which positions working memory and attention control as regulatory mechanisms that shape vocabulary retention during conversational AI interactions.

**H1.**  *Working memory positively predicts attention control in ChatGPT-assisted foreign language learning.*

**H2.**  *Working memory positively predicts vocabulary retention.*

**H3.**  *Attention control positively predicts vocabulary retention.*

**H4.**  *Attention control mediates the relationship between working memory and vocabulary retention.*

**H5.**  *Frequency of ChatGPT use moderates the working memory to attention control relationship, such that the association is stronger at higher frequency of use.*

**H6.**  *Frequency of ChatGPT use moderates the attention control to vocabulary retention relationship, such that the association is stronger at higher frequency of use.*

**Figure 1:**
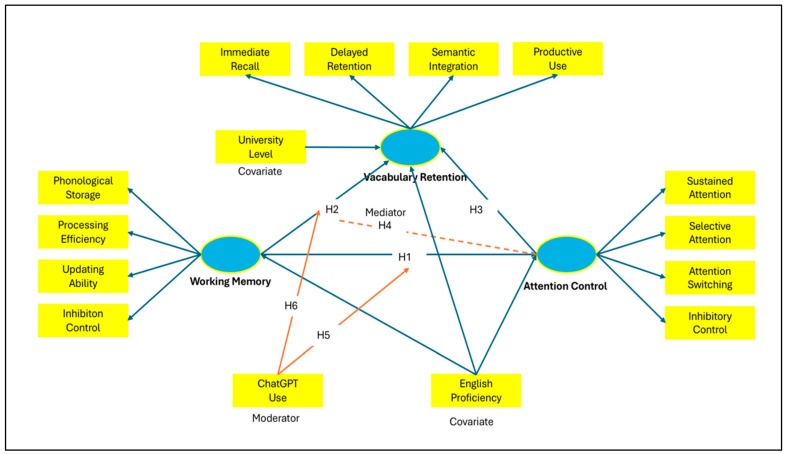
Hypothesised Structural Model of Working Memory, Attention Control, and Vocabulary Retention in ChatGPT-Assisted Foreign Language Learning.

Figure 1 illustrates the hypothesised structural model underpinning the study. Working memory and attention control are specified as core cognitive predictors of multidimensional vocabulary retention, with attention control positioned as a mediating mechanism and frequency of ChatGPT use modelled as a moderator of selected pathways. English proficiency and university level are included as covariates. Based on this model, the hypotheses below define the proposed direct, indirect, and conditional relationships among the study variables.

## 3. Research Methods

### 3.1. Research Design

This study adopted a quantitative cross-sectional correlational survey design to examine the structural relationships among working memory, attention control, frequency of ChatGPT use, and vocabulary retention in foreign language learning. The design was used to test a theoretically specified structural model through structural equation modelling (SEM), allowing examination of direct associations, mediated relationships, and moderated pathways within a single analytical framework.

### 3.2. Participant Recruitment

The study sample comprised 1002 university students enrolled in English-related courses across three public universities. A stratified random sampling strategy was employed to secure proportional representation across institutions and academic levels. The population within each university was first stratified by year of study, after which participants were randomly selected within each stratum. This procedure reduced sampling bias while preserving variability in academic experience relevant to the proposed cognitive model. Eligibility was restricted to students with prior experience using ChatGPT for English learning activities to ensure that responses reflected actual engagement with conversational AI.

As reported in Table 1, the gender distribution was relatively balanced, with 538 male participants (53.7%) and 464 female participants (46.3%). Participants covered a broad age range, with most aged 18–26 (77.1%), consistent with undergraduate populations in public higher education contexts. Academic standing was well distributed across levels. Third-year students constituted the largest group (28.8%), followed by second-year students (27.0%), first-year students (22.6%), and fourth-year or higher students (21.6%). This distribution provided sufficient dispersion in academic experience to warrant the inclusion of university-level experience as a covariate in the structural analyses. Reported patterns of ChatGPT use indicated sustained engagement with the tool. A majority of participants reported using ChatGPT often (35.5%) or sometimes (31.7%), while 18.4% indicated very frequent use and 14.4% reported infrequent use. Self-rated English proficiency ranged from beginner to advanced, with most participants identifying as intermediate (41.7%) or upper-intermediate (30.7%), supporting its treatment as a control variable in modelling cognitive predictors of vocabulary retention.

From a statistical perspective, the final sample size exceeded recommended thresholds for SEM with latent constructs, mediation, and moderation paths. Methodological guidance for complex SEM designs indicates that large samples are required to obtain stable parameter estimates and adequate power. With more than 1000 observations, the present study was sufficiently powered to detect medium-sized direct and indirect effects while maintaining robust model estimation. The combination of multi-institutional sampling, random selection within strata, and substantial sample size provides a strong empirical basis for testing the hypothesised relationships among working memory, attention control, frequency of ChatGPT use, and multidimensional vocabulary retention in public university EFL contexts.

### 3.3. Instruments

Data were collected using a structured self-report questionnaire designed to measure working memory, attention control, and vocabulary retention in ChatGPT-assisted foreign language learning. Because these constructs were operationalised through self-report items rather than behavioural cognitive tasks or objective vocabulary tests, the measures reflect learners’ reported cognitive regulation and perceived vocabulary retention during ChatGPT-assisted learning rather than direct assessments of cognitive capacity or actual retention performance, as self-report measures are widely used to capture perceived cognitive regulation and learning processes in technology-mediated language contexts (Hao et al., 2021; Teng & Zhang, 2021). All items were framed with explicit reference to learners’ engagement with ChatGPT, anchoring responses in actual learning behaviour rather than general ability judgments. Responses were recorded on a five-point Likert scale ranging from 1 (strongly disagree) to 5 (strongly agree) (see Supplementary Materials).

The questionnaire consisted of two sections. The first section gathered demographic and contextual information, including gender, age group, university level, frequency of ChatGPT use, and self-rated English proficiency. These variables were used to characterise the sample and to specify control and moderating variables in the structural model.

The second section comprised 48 items distributed evenly across three latent constructs, each operationalised through four theoretically grounded dimensions. Working memory was measured using 16 items that reflected phonological storage, processing efficiency, updating ability, and inhibitory control. This specification aligns with cognitive accounts that link lexical learning to the temporary maintenance, revision, and regulation of linguistic representations under interference during language use (Bialystok & Craik, 2022; Friedman & Robbins, 2021), as well as evidence from language processing research showing that working memory supports the maintenance and updating of competing lexical representations during comprehension and production (Fedorenko & Shain, 2021; Shain et al., 2024). In this context, inhibition refers specifically to interference control during the maintenance and updating of lexical representations, that is, the ability to protect task-relevant linguistic information in working memory while filtering out competing lexical alternatives that arise during interaction.

Attention control was assessed using 16 items that captured sustained attention, selective attention, attentional switching, and inhibitory control. These dimensions reflect established evidence that successful lexical processing in interactive environments depends on learners’ capacity to regulate focus, prioritise relevant input, and manage shifts between comprehension and production under cognitive load (Ellis, 2019; Qiu et al., 2025), with additional support from research showing that attentional regulation governs the selection and suppression of competing linguistic information during interactive language use (Czerwińska & Piskorska, 2018). Within this construct, inhibitory control refers to attentional suppression during task engagement, including the regulation of distracting prompts, irrelevant suggestions, or competing task demands during ChatGPT interaction. Although both constructs involve inhibitory regulation, the questionnaire differentiates between interference control operating within working memory processes and attentional suppression governing task-level focus and behavioural regulation.

Vocabulary retention was measured using 16 items representing immediate recall, delayed retention, semantic integration, and productive use. This multidimensional structure follows empirical work showing that vocabulary knowledge develops across temporally and functionally distinct memory processes rather than as a single outcome (Teng, 2022; Webb et al., 2023), with further evidence indicating that these dimensions reflect distinct retention processes shaped by encoding conditions, consolidation, and retrieval demands (Hao et al., 2021; Teng & Zhang, 2023).

All items were reviewed for clarity, construct relevance, and contextual specificity prior to administration. The multidimensional structure of each construct was specified a priori to support latent-variable modelling and examination of differentiated cognitive pathways linking working memory, attention control, and vocabulary retention outcomes. Detailed procedures for scale validation and model verification are reported in the following subsection.

#### Instrument Validation Procedures

Instrument validation followed a staged procedure integrating expert review and latent-structure verification. Prior to large-scale administration, the initial item pool was examined by specialists in applied linguistics, cognitive psychology, and language assessment to assess construct alignment, linguistic clarity, and contextual suitability for ChatGPT-assisted learning. Minor wording refinements were introduced to improve precision while preserving the theoretical scope of each item.

For empirical validation, the dataset was randomly partitioned to allow independent examination of measurement structure and subsequent structural analysis. A total of 650 responses were allocated to scale validation. Exploratory factor analysis (EFA) was conducted on 300 responses to examine the latent configuration of the working memory, attention control, and vocabulary retention scales. Principal axis factoring with oblique rotation was applied, reflecting the expectation that cognitive dimensions are correlated. Item retention decisions were based on factor loadings, cross-loading behaviour, and conceptual consistency with the predefined dimensions.

Confirmatory factor analysis (CFA) was performed on an independent subsample of 350 responses to test the factor structures identified through EFA. Model adequacy was evaluated using established goodness-of-fit indices reported in SEM research, including the comparative fit index, Tucker–Lewis index, root mean square error of approximation, and standardised root mean square residual. Standardised factor loadings supported the specified measurement models, indicating satisfactory correspondence between observed indicators and their intended latent constructs.

Internal consistency was assessed using composite reliability coefficients, while convergent validity was evaluated through average variance extracted values. Discriminant validity was examined by comparing the square root of the average variance extracted for each construct with its correlations with other constructs. Following confirmation of acceptable measurement properties, the full sample (N = 1002) was used to estimate the structural equation model, testing the hypothesised direct, mediated, and moderated relationships among working memory, attention control, frequency of ChatGPT use, and multidimensional vocabulary retention outcomes.

### 3.4. Data Collection and Ethical Considerations

Data were gathered during the first term of the 2025–2026 academic year across the three participating public universities, with collection extending over a two-month period. The questionnaire was administered online via Qualtrics, which provided a controlled environment for anonymous responses and secure data management. Course instructors assisted with dissemination by sharing the survey link with eligible students enrolled in English-related courses. Participants completed the questionnaire at their convenience, independent of graded course activities. Prior to participation, students were presented with a detailed information statement outlining the study objectives, the voluntary nature of participation, and their rights as participants. Access to the questionnaire was contingent on providing electronic informed consent. The survey did not request personal identifiers, and all responses were treated as confidential. Ethical clearance for the study was obtained from Ha’il University prior to data collection. Data handling procedures adhered to established ethical standards for educational research, with all records stored in secure, password-protected files and accessible only to the research team. Given that the study involved self-report measures related to routine learning practices, it posed minimal risk to participants and complied with relevant institutional and national research ethics requirements.

### 3.5. Data Analysis

Prior to the main analyses, the dataset was screened for missing values, outliers, and distributional assumptions. Missing data were minimal and were handled using listwise deletion. Skewness and kurtosis values fell within acceptable ranges, and multicollinearity diagnostics indicated no problematic correlations among the study variables. All analyses were conducted using IBM SPSS 26 Statistics and AMOS 26. Data were analysed using IBM SPSS Statistics and AMOS following a sequential procedure appropriate for latent-variable modelling. Descriptive statistics and zero-order correlations were first computed in SPSS to summarise the distributional characteristics of the study variables and examine preliminary associations among working memory, attention control, frequency of ChatGPT use, and vocabulary retention (see Supplementary Materials). EFA was then conducted to examine the latent structure of the measurement instruments, followed by CFA in AMOS to verify the adequacy of the measurement models prior to structural testing. After an acceptable measurement fit was established, SEM was performed in AMOS to test the hypothesised direct relationships, the mediating role of attention control, and the moderating role of frequency of ChatGPT use. Indirect effects were examined using bias-corrected bootstrap confidence intervals based on 5000 resamples, while moderation was assessed through latent interaction terms. Model adequacy was evaluated using standard SEM fit criteria, and statistically significant interaction effects were interpreted through simple slope analyses, with significance evaluated at the 0.05 level.

## 4. Findings

### 4.1. Exploratory Factor Analysis

EFA was undertaken to examine the latent structure of the working memory, attention control, and vocabulary retention scales prior to confirmatory modelling. As reported in Table 2, sampling adequacy was supported by Kaiser–Meyer–Olkin values ranging from 0.81 to 0.84, indicating sufficient shared variance among items. Bartlett’s test of sphericity was statistically significant for all three scales (χ^2^ = 1214.47–1948.63, df = 120, *p* < .001), confirming that the correlation matrices were suitable for factor extraction. Based on these results, factor analysis was conducted separately for each construct using principal axis factoring with oblique rotation, consistent with the expectation that cognitive dimensions are correlated.

Following the assessment of sampling adequacy and factorability reported in Table 2, EFA was conducted for the three scales. The analysis yielded a four-factor solution for each construct, consistent with the proposed dimensional structures of working memory, attention control, and vocabulary retention. The retained factors accounted for 68.94% of the variance in working memory, 69.44% in attention control, and 70.07% in vocabulary retention, indicating that a substantial proportion of shared variance was captured across all scales. Item loadings were satisfactory overall, with a small number of items removed due to weak or cross-loading patterns. Inspection of the scree plots supported retaining four factors for each construct, with clear inflexion points after the fourth component. These results provide consistent support for the specified factor structures.

The rotated solution for the working memory scale produced a clear four-factor structure corresponding to phonological storage, processing efficiency, updating ability, and inhibition control. Items were loaded as expected into their respective components, with minimal cross-loading. Two items did not meet the 0.40 loading criterion and showed poor differentiation across factors and were therefore removed. Their exclusion improved the clarity of the factor structure without altering the construct’s conceptual coverage.

A comparable pattern emerged for the attention control scale. The four components, sustained attention, selective attention, attention switching, and inhibitory control, were well differentiated, with items loading consistently on their intended dimensions. One item showed weak alignment with the underlying structure and was removed, resulting in a more coherent representation of the construct.

For vocabulary retention, the rotated solution likewise supported a four-factor structure reflecting immediate recall, delayed retention, semantic integration, and productive use. Most items aligned well with their respective dimensions. Four items did not integrate satisfactorily within the factor structure and were removed. The revised solution provided a clearer and more stable representation of vocabulary retention for subsequent analysis. Detailed EFA results, including rotated component matrices, are reported in the Supplementary Materials.

### 4.2. Confirmatory Factor Analysis

The adequacy of the measurement models was evaluated through CFA, with fit indices reported in Table 3. The results indicate satisfactory model fit for all three constructs. The χ^2^/df ratios were below 2.00 for working memory, attention control, and vocabulary retention, supporting acceptable parsimony. Incremental fit indices exceeded recommended benchmarks, with CFI values ranging from 0.955 to 0.962 and TLI values from 0.946 to 0.954, indicating strong correspondence between the hypothesised models and the observed data. Absolute fit indices further supported model adequacy, as RMSEA values remained below 0.05 and SRMR values were well within acceptable limits across all constructs. These results support the adequacy of the specified measurement models and justify proceeding to structural model estimation. In addition to the construct-level CFAs reported above, the structural equation modelling procedure estimated the latent constructs simultaneously within a single model. Because SEM estimates measurement and structural components concurrently, the adequacy of the combined latent structure was evaluated through the overall model fit indices reported for the structural model (see Figure 6 and Table 7). The satisfactory fit of the structural model, therefore, provides evidence that the full latent structure underlying the mediation and moderation paths was adequately represented in the data, supporting the validity of the combined measurement framework prior to interpretation of the structural relationships.

Convergent and discriminant validity were evaluated using Cronbach’s alpha (α), composite CR, AVE, and inter-factor correlations, as reported in Table 4. Internal consistency was satisfactory across all subscales, with α values ranging from 0.75 to 0.85 and CR values from 0.75 to 0.86, indicating stable measurement. AVE values exceeded 0.50 for all dimensions (range = 0.50–0.61), supporting adequate convergence of indicators within each latent construct. Discriminant validity was supported as the square roots of AVE (ranging from 0.71 to 0.78) exceeded the corresponding inter-factor correlations for all subscales, confirming adequate separation among the dimensions of working memory, attention control, and vocabulary retention.

**Figure 2:**
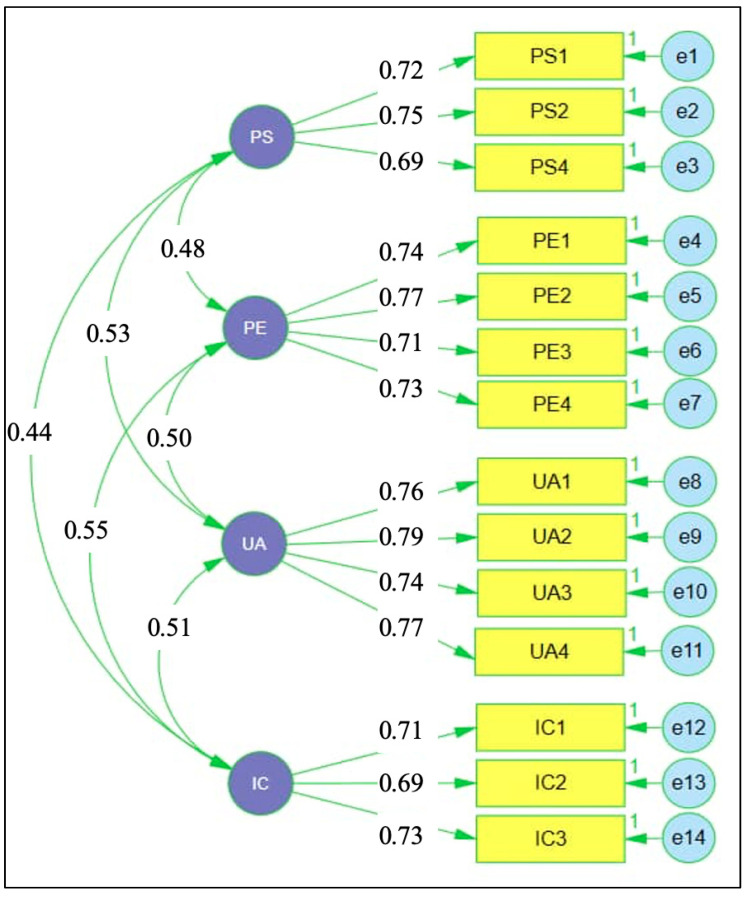
CFA for Working Memory Scale.

Figure 2 presents the CFA results for the working memory scale. Standardised factor loadings were substantial, ranging from 0.69 to 0.79, indicating strong associations between observed indicators and their respective latent dimensions of phonological storage, processing efficiency, updating ability, and inhibition control. Correlations among the latent factors were moderate, ranging from 0.44 to 0.55, indicating related but empirically distinguishable components within the broader working memory construct. This pattern supports the adequacy of the specified measurement model and confirms that the retained indicators provide a stable representation of working memory for subsequent structural analysis.

**Figure 3:**
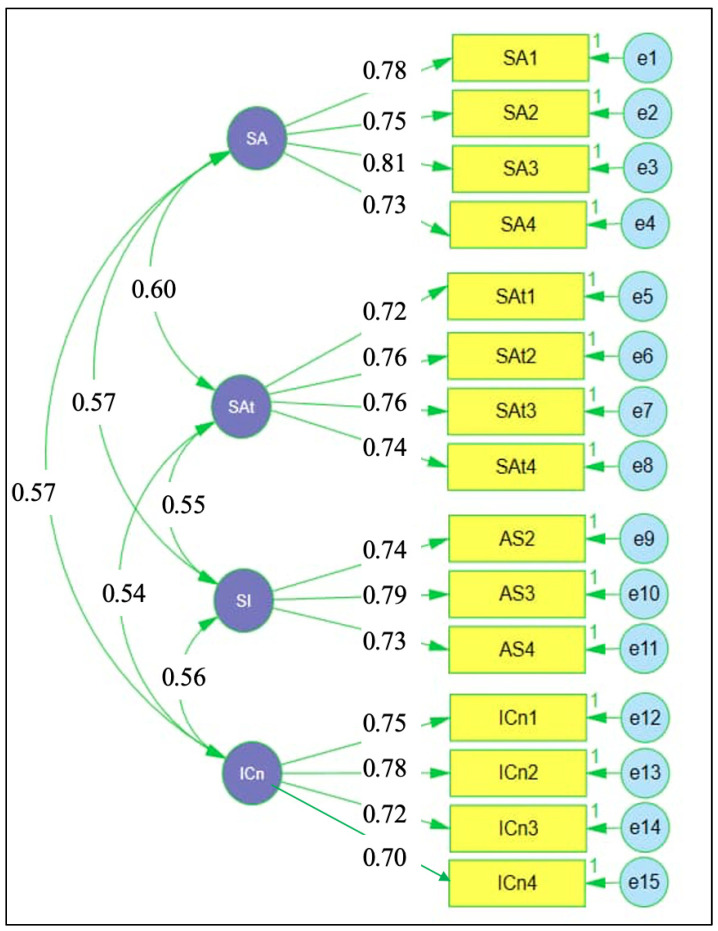
CFA for Attention Control Scale.

Figure 3 illustrates the CFA results for the attention control scale. Standardised factor loadings across the four dimensions, sustained attention, selective attention, attention switching, and inhibitory control, were consistently strong, ranging from 0.70 to 0.81, indicating robust associations between observed indicators and their respective latent constructs. Correlations among the latent dimensions were moderate, with coefficients ranging from 0.54 to 0.60, reflecting related but empirically distinguishable components of attention regulation. This pattern supports the adequacy of the specified measurement model and confirms that the retained indicators provide a stable and coherent representation of attention control for subsequent structural analysis.

**Figure 4:**
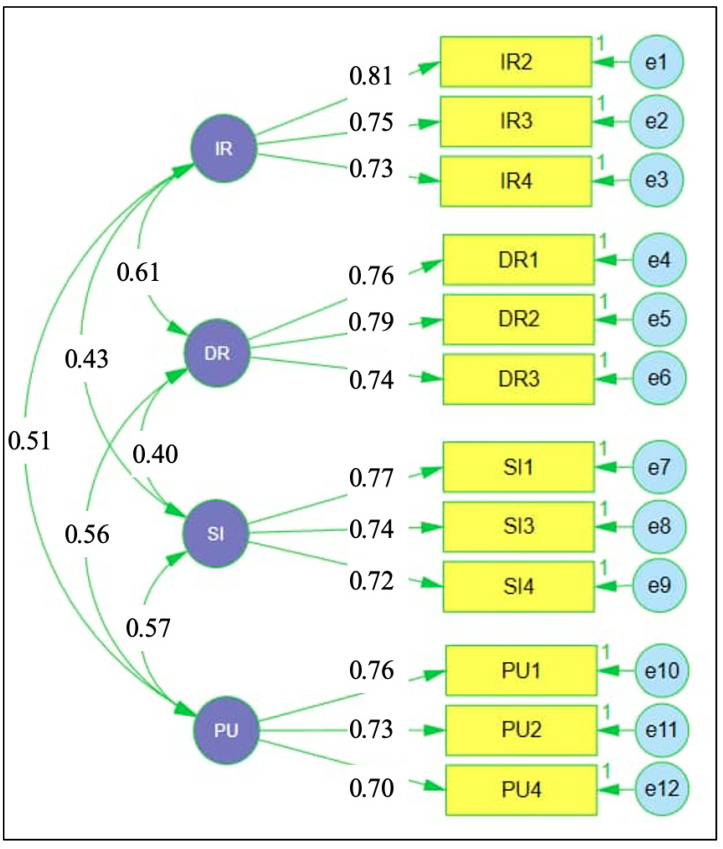
CFA for Vocabulary Retention Scale.

Figure 4 presents the CFA results for the vocabulary retention scale. Standardised factor loadings across the four dimensions, immediate recall, delayed retention, semantic integration, and productive use, were substantial, ranging from 0.70 to 0.81, indicating strong associations between observed indicators and their respective latent factors. Correlations among the latent dimensions were moderate, with coefficients ranging from 0.40 to 0.61, reflecting related yet empirically distinct components of vocabulary retention. This pattern supports the adequacy of the specified measurement model and confirms that the retained indicators provide a stable representation of differentiated vocabulary retention outcomes for subsequent structural analysis.

Potential common method variance was assessed using the common latent factor (CLF) technique, with results summarised in Table 5. Comparisons of standardised loadings before and after inclusion of the common latent factor showed very small differences, ranging from 0.01 to 0.03 across all indicators for working memory, attention control, and vocabulary retention. These minimal changes indicate that the variance attributable to a common measurement source was negligible and did not materially alter the strength of the item–construct relationships. On this basis, common method bias is unlikely to compromise the validity of the measurement model.

### 4.3. Preliminary Measurement Evaluation

**Figure 5:**
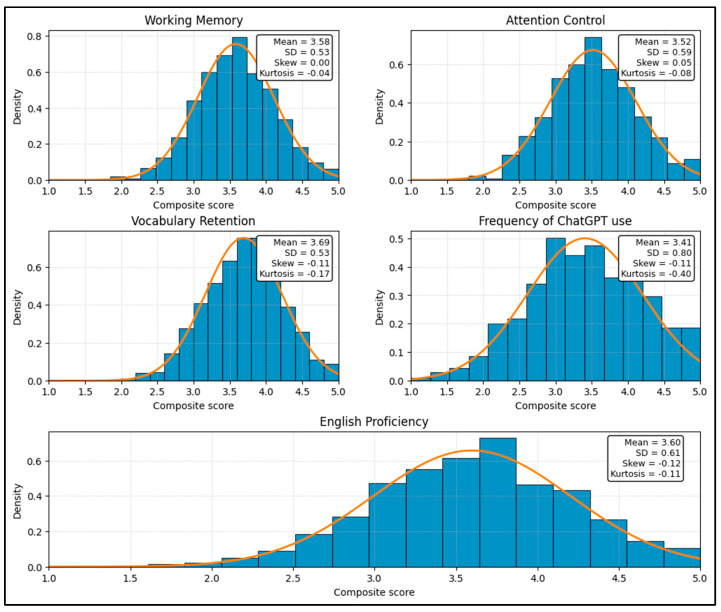
Histogram with normality curves.

Preliminary diagnostics were conducted to examine distributional properties and collinearity prior to structural model estimation. As shown in Figure 5, the composite scores for working memory, attention control, vocabulary retention, frequency of ChatGPT use, and English proficiency exhibited approximately normal distributions. Descriptive indices indicated moderate central tendency (means ranging from 3.41 to 3.69) and acceptable dispersion (standard deviations between 0.53 and 0.80). Skewness values were close to zero (−0.12 to 0.05) and kurtosis values were modest (−0.40 to −0.04), supporting the assumption of univariate normality for subsequent SEM analyses.

Multicollinearity diagnostics for predictors included in the structural model are reported in Table 6. Variance inflation factor (VIF) values ranged from 1.17 to 2.22, with corresponding tolerance values between 0.45 and 0.85, indicating no evidence of problematic collinearity among predictors. Interaction terms involving the frequency of ChatGPT use also remained within acceptable limits (VIF ≤ 2.22), suggesting that moderation effects could be estimated without inflated standard errors. These results indicate that the data satisfied key distributional and independence assumptions required for reliable estimation of the structural model.

### 4.4. Hypothesis Testing

**Figure 6:**
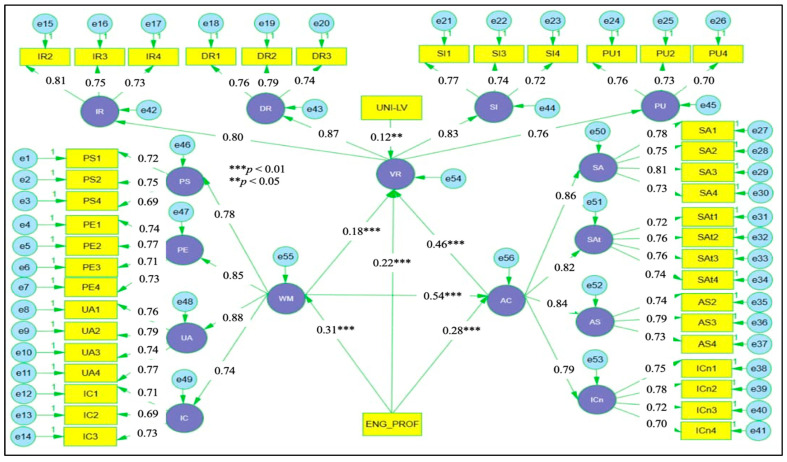
SEM path model.

The hypothesised relationships were examined through SEM, with the estimated paths presented in Figure 6. Working memory showed a strong positive association with attention control (β = 0.54, *p* < .001), indicating that learners with greater capacity to maintain, update, and regulate information were more effective in sustaining and directing attention during ChatGPT-assisted tasks. This result supports H1. Working memory also displayed a direct positive relationship with vocabulary retention (β = 0.22, *p* < .001), supporting H2 and indicating that cognitive capacity contributes to lexical retention beyond attentional regulation alone.

Attention control was a substantial predictor of vocabulary retention (β = 0.46, *p* < .001), supporting H3. This finding indicates that learners’ ability to sustain focus, prioritise relevant input, shift efficiently between comprehension and production, and inhibit distraction plays a central role in retaining vocabulary acquired through conversational interaction. Mediation analysis showed that attention control had a significant indirect effect from working memory to vocabulary retention (β = 0.18, *p* < .001), supporting H4. The presence of both direct and indirect effects indicates partial mediation, suggesting that working memory contributes to vocabulary retention through attentional regulation and direct support for lexical processing.

Moderation analyses further clarified the role of engagement intensity. The interaction between working memory and frequency of ChatGPT use significantly predicted attention control (β = 0.31, *p* < .001), supporting H5. This pattern indicates that frequent engagement with ChatGPT strengthens the association between cognitive capacity and attentional regulation. In parallel, the interaction between attention control and frequency of ChatGPT use was a significant predictor of vocabulary retention (β = 0.28, *p* < .001), supporting H6 and indicating that attentional regulation translated more effectively into retention outcomes when learners engaged with the system more regularly.

Among the control variables, English proficiency was positively associated with vocabulary retention (β = 0.28, *p* < .001), while university level showed a smaller but statistically significant effect (β = 0.12, *p* < .05). Overall, the structural results demonstrate that vocabulary retention in ChatGPT-assisted foreign language learning is shaped by a coordinated pattern of cognitive capacity, attentional regulation, and engagement frequency, with attention control functioning as a key pathway linking working memory to differentiated retention outcomes.

The structural estimates reported in Table 7 confirm the adequacy of the hypothesised model. Working memory showed a strong association with attention control (β = 0.54, *p* < .001), while attention control was the dominant predictor of vocabulary retention (β = 0.46, *p* < .001). The indirect pathway linking working memory to vocabulary retention through attention control was statistically robust (β = 0.25, *p* < .001), indicating that attentional regulation accounted for a substantial share of the cognitive effect. Moderation analyses indicated that frequency of ChatGPT use conditioned both cognitive pathways, strengthening the working memory–attention control link (β = 0.14, *p* = .001) and the attention control–vocabulary retention link (β = 0.17, *p* = .002). Covariate effects showed that English proficiency contributed consistently across cognitive and outcome variables, while university level exerted a smaller effect, limited to vocabulary retention.

**Figure 7:**
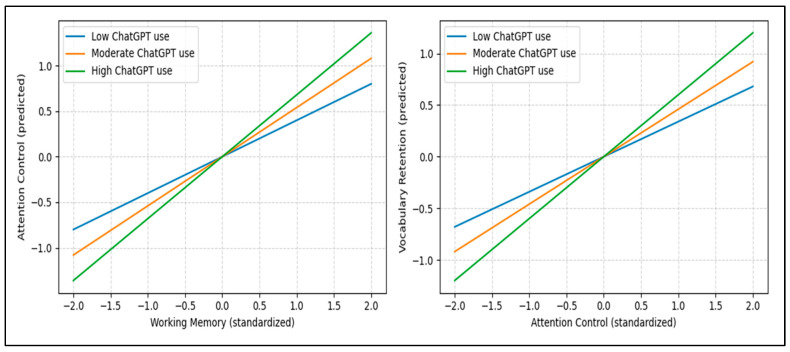
Simple slopes explaining the moderation effects.

The moderation patterns depicted in Figure 7 complement the structural estimates by clarifying how engagement intensity conditions the cognitive pathways under examination. The simple slopes indicate that a higher frequency of ChatGPT use amplifies the extent to which working memory supports attentional regulation and, in turn, the extent to which attention control translates into vocabulary retention. At lower levels of engagement, these relationships remain positive but comparatively attenuated, suggesting that cognitive resources are less fully translated into learning outcomes when interaction with the system is limited. This pattern reinforces the interpretation that conversational AI functions as a context that can strengthen existing cognitive capacities rather than substitute for them, with the frequency of use shaping the extent to which working memory and attentional control are mobilised during vocabulary learning.

## 5. Discussion

### 5.1. Cognitive Capacity in ChatGPT-Assisted Vocabulary Learning (H1, H2)

The findings related to H1 and H2 indicate that working memory is strongly associated with foreign language vocabulary learning mediated by ChatGPT. The structural relationships underlying these findings are illustrated in Figure 7, which presents the final structural model based on the SEM results. Working memory showed a strong positive association with attention control (H1) and a positive association with vocabulary retention (H2), indicating that learners with greater capacity to maintain, update, and regulate information tended to sustain attentional focus and retain newly learned foreign language lexical items during conversational interaction. These results suggest that, within foreign language learning contexts, conversational AI environments do not neutralise individual cognitive differences; rather, they appear to make such differences consequential for learning outcomes.

The relationship proposed in H1 can be interpreted in light of the specific processing demands of second- and foreign-language interaction. When engaging with ChatGPT in a non-native language, learners must decode unfamiliar lexical forms, evaluate alternative wordings, and maintain semantic coherence while planning responses. These demands require the temporary maintenance of task-relevant representations amid lexical competition and interference. Working memory capacity is likely to support this process by allowing learners to keep multiple L2 lexical candidates active while directing attention toward forms and meanings that remain relevant for the ongoing exchange. In this context, working memory may function as a regulatory cognitive resource associated with attentional control during foreign language processing, rather than operating solely as a passive storage system (Bialystok & Craik, 2022; Friedman & Robbins, 2021).

The direct association proposed in H2 suggests that working memory is related to foreign language vocabulary retention beyond its role in attentional regulation. Conversational interaction in a non-native language requires learners to rehearse, revise, and integrate new lexical items while discourse unfolds, placing sustained pressure on updating and interference management. Learners with greater working memory capacity appear to be better able to stabilise L2 form–meaning representations during these exchanges, which may support retention even when attentional processes are taken into account. This interpretation aligns with cognitive accounts of second-language lexical learning that frame retention as dependent on the ability to maintain and reorganise linguistic information under processing load (Fedorenko & Shain, 2021; Luque & Morgan-Short, 2021).

These findings align with prior research in foreign language learning, which shows that working memory capacity is associated with both immediate and longer-term vocabulary retention under conditions of high cognitive demand, particularly in technology-mediated and task-based contexts (Hao et al., 2021; Teng & Zhang, 2021). Research on second-language writing and multimedia learning further indicates that working memory is related to lexical accuracy and retention when learners must manage competing information streams and continuous production demands in a non-native language (Kormos, 2023; Li, 2023). The present study adds evidence from conversational AI environments, suggesting that similar cognitive constraints may be present in foreign-language interaction mediated by generative systems.

At the same time, these results diverge from studies that attribute vocabulary gains in ChatGPT-assisted foreign language learning primarily to increased exposure or system responsiveness. Although prior work frequently reports positive learner outcomes, cognitive processing is often treated implicitly or assumed to be secondary (AlTwijri & Alghizzi, 2024; Xu et al., 2024; Zaim et al., 2025). The current evidence suggests that exposure alone may not fully explain patterns of foreign-language vocabulary retention. Instead, working memory capacity appears to influence learners’ ability to manage lexical density and sustain learning-relevant representations during non-native language interaction. This position aligns with cognitive and usage-based perspectives that treat foreign language learning as constrained by processing resources rather than driven solely by input availability (Ellis, 2019; Shain et al., 2024).

In sum, the findings supporting H1 and H2 suggest that ChatGPT-assisted foreign-language vocabulary learning remains cognitively demanding and capacity-sensitive. Generative conversational systems do not replace internal cognitive resources; rather, they appear to create interactional conditions in which working memory capacity is associated with attentional regulation and lexical retention during second- and foreign-language use.

### 5.2. Attention Control as the Regulatory Mechanism of Vocabulary Retention (H3, H4)

The findings for H3 and H4 suggest that attention control is strongly related to the relationship between cognitive capacity and foreign-language vocabulary retention in ChatGPT-assisted learning. Attention control showed a strong positive association with vocabulary retention (H3) and accounted for a substantial portion of the association between working memory and retention outcomes (H4). These results suggest that successful vocabulary learning in conversational AI environments is closely related to learners’ ability to regulate focus, prioritise relevant lexical information, and maintain task engagement during non-native language interaction.

The relationship proposed in H3 can be interpreted in light of the attentional demands imposed by conversational foreign-language use. Interaction with ChatGPT requires learners to alternate rapidly between comprehension and production while processing dense lexical input. Such conditions increase susceptibility to distraction, reformulation overload, and premature topic shifts, particularly in a non-native language. Attention control appears to support vocabulary retention by helping learners select learning-relevant lexical items, sustain focus across conversational turns, and suppress competing or redundant alternatives. In foreign-language contexts, these regulatory processes may influence which lexical forms receive sufficient cognitive processing to support consolidation, rather than remaining transient elements of interaction (Czerwińska & Piskorska, 2018; Qiu et al., 2025).

The mediating role observed in H4 suggests that attention control may represent the pathway through which working memory capacity is associated with durable vocabulary knowledge. While working memory supports the maintenance of multiple representations, attention control appears to shape how these representations are prioritised and stabilised during learning. Research in second- and foreign-language processing shows that lexical competition and variability during interaction exert pressure on attentional selection, particularly when learners must discriminate among alternative forms and meanings under time constraints (Parris et al., 2021; Zhu et al., 2021). In conversational AI settings, learners are exposed to frequent paraphrasing and lexical reformulation, which can fragment processing unless attentional regulation helps maintain focus on form–meaning relations that remain instructionally relevant. Evidence from interactive and multimedia vocabulary learning further indicates that retention is associated with the extent to which attentional resources are directed toward lexical encoding rather than dispersed across competing stimuli (Teng, 2022; Webb et al., 2023). Mediation in this context, therefore, should be interpreted as a statistical pattern consistent with a regulatory process rather than as evidence of causal mediation: working memory is associated with attentional regulation, and attentional regulation is, in turn, associated with whether lexical information is retained beyond the immediate interaction.

Evidence from research on foreign-language vocabulary supports this regulatory interpretation of attention control. Studies conducted in interactive and multimedia learning contexts show that vocabulary retention is associated with learners’ ability to allocate attention effectively during encoding rather than with exposure volume alone, with stronger retention observed when tasks require sustained focus on lexical form–meaning relations and active output (Teng, 2022; Webb et al., 2023). Research in augmented and immersive learning environments further shows that the direction of attention during interaction is related to both immediate and delayed vocabulary outcomes, indicating that attentional regulation is linked to the depth of lexical processing achieved during learning (Yilmaz et al., 2022). Converging evidence from AI-supported language learning reinforces this position, as studies on AI-driven feedback report stronger vocabulary gains when feedback channels learners’ attention toward moments of lexical retrieval and production instead of passive exposure (Tangpijaikul, 2025). This pattern aligns with findings from spoken interaction research showing that attentional control is associated with lexical accuracy and retention through its role in regulating retrieval demands during communicative use (Parris et al., 2021). In contrast to studies that treat vocabulary retention as an automatic consequence of conversational engagement, the present findings associated with H3 and H4 suggest that attentional regulation is closely linked to foreign language learning in AI-mediated environments, indicating that attention control may influence whether conversational input is associated with retained lexical knowledge under conditions of high informational density and continuous interaction.

### 5.3. Frequency of ChatGPT Use as a Conditioning Context (H5, H6)

The findings related to H5 and H6 suggest that the frequency of ChatGPT use is linked to the relationship between working memory and attention control and foreign language vocabulary retention. Higher levels of engagement strengthened the association between working memory and attention control (H5) and were associated with a stronger relationship between attention control and vocabulary retention (H6). These results indicate that frequency of use does not operate as an independent driver of vocabulary learning; rather, it appears to shape the interactional conditions under which cognitive resources are mobilised during sustained foreign language interaction.

One explanation may lie in the role of repeated engagement in reducing non-linguistic processing demands during technology-mediated learning. As learners interact with ChatGPT more frequently, they become familiar with the structure of conversational exchanges, the pacing of system responses, and the form of feedback provided. This familiarity may lessen the cognitive effort required for task management and interface navigation, allowing cognitive resources to be directed more consistently toward language processing. Research on computer-assisted and game-based language learning shows that repeated exposure to digital environments is associated with more stable attentional regulation and stronger vocabulary retention once learners no longer need to devote cognitive effort to managing task mechanics (Ahmed et al., 2022; Kazu & Kuvvetli, 2023). From this perspective, frequency of ChatGPT use may influence the efficiency with which working memory supports attentional regulation during lexical processing.

The moderation observed in H6 further suggests that attention control is more strongly associated with vocabulary retention when learners engage with ChatGPT regularly. Sustained interaction creates additional opportunities for learners to regulate their attention during lexical retrieval, reformulation, and use, particularly in output-oriented activities. Evidence from studies of ASR-based and chatbot-supported language learning indicates that vocabulary development is more consistent when learners repeatedly practise lexical production with guided technological support, allowing attentional regulation to stabilise across communicative episodes (Bashori et al., 2021, 2022; Z. Zhang & Huang, 2024). Related findings from interactive task research also show that repeated engagement is associated with learners’ ability to manage cognitive demands during foreign language production, resulting in more reliable lexical access over time (Qiu et al., 2025).

Compared with research that treats frequency of technology use as a descriptive usage indicator, the present findings frame engagement intensity as a contextual factor associated with cognitive regulation during foreign language learning. Studies examining multimedia input, mobile-assisted learning, and out-of-class vocabulary practice report that repeated engagement is linked to stronger retention primarily when it encourages sustained attentional focus and controlled lexical processing rather than passive exposure (Zakian et al., 2022; Teng & Zhang, 2023). In ChatGPT-assisted learning, the results for H5 and H6 suggest that frequent interaction coincides with stronger coordination between working memory and attention control during non-native language use. In this sense, conversational AI can be understood as an interactional environment that redistributes cognitive demands across repeated encounters rather than a tool that produces learning gains through frequency of use alone.

Although the present model focuses on working memory and attention control as central regulatory resources in ChatGPT-assisted vocabulary learning, vocabulary development in foreign-language contexts is likely supported by a broader range of cognitive mechanisms, including retrieval processes, consolidation dynamics, and semantic memory systems. Therefore, the current analysis isolates two core regulatory processes to clarify their structural contributions to vocabulary retention in conversational AI interactions.

## 6. Theoretical and Practical Implications

From a theoretical perspective, the findings refine current accounts of ChatGPT-assisted foreign-language vocabulary learning by demonstrating that learning outcomes are shaped by cognitive regulation rather than by interactional exposure alone (Ellis, 2019). Working memory contributes to vocabulary retention both directly and by supporting attention control, while attention control serves as the mechanism that determines whether conversational input is transformed into retained lexical knowledge. The moderation effects further show that the frequency of ChatGPT use conditions the operation of these cognitive pathways rather than acting as an independent predictor of learning. These results support a process-oriented view of AI-mediated language learning in which conversational systems function as cognitively demanding environments that amplify individual differences in regulatory capacity. The model extends cognitive approaches to second language acquisition by specifying how working memory, attention control, and engagement intensity jointly structure vocabulary learning in conversational AI contexts (Bialystok & Craik, 2022). Although the model focuses on two regulatory mechanisms, vocabulary development in foreign-language contexts likely involves additional processes, such as retrieval operations, consolidation dynamics, and semantic memory systems. The present analysis isolates working memory and attention control to clarify their structural contributions to vocabulary retention during conversational AI interactions, while maintaining conceptual focus within a tractable cognitive framework.

From a practical perspective, the findings indicate that the effectiveness of ChatGPT-assisted vocabulary learning depends on how learners regulate attention during interaction and how consistently they engage with the system over time. Instructional use of ChatGPT should therefore prioritise task designs that guide learners’ attentional focus toward lexical form–meaning relations during interaction rather than relying on conversational exposure alone (Teng & Zhang, 2023). Regular, structured engagement appears necessary to allow learners to allocate cognitive resources more efficiently as interactional routines stabilise. These results suggest that pedagogical value lies not in increasing the volume of AI interaction, but in shaping conditions under which working memory and attention control can be deployed effectively during foreign language use. Consequently, instructional integration of ChatGPT should be framed as a cognitively regulated learning activity rather than a self-directed conversational supplement.

## 7. Limitations and Recommendations for Future Studies

One limitation of the present study lies in the scope of the cognitive model, which focused on working memory and attention control as the primary regulatory processes shaping ChatGPT-assisted vocabulary retention, leaving other relevant mechanisms such as inhibitory control, cognitive flexibility, or metacognitive monitoring outside the analytical framework as well as learning processes associated with retrieval practice, consolidation, semantic integration, and distinctions between implicit and explicit learning conditions that may contribute to the stabilisation of lexical knowledge over time (Teng, 2022; Webb et al., 2023); future studies should extend the model to examine whether these additional regulatory processes contribute independently or interact with attention control during conversational AI-mediated foreign language learning. A second limitation concerns the measurement of cognitive constructs through self-report instruments. Such measures capture learners’ perceptions of their cognitive functioning during ChatGPT-assisted learning rather than the cognitive processes themselves, and may therefore partly reflect metacognitive awareness of cognitive regulation. Accordingly, the constructs examined in this study should be interpreted as indicators of learners’ reported cognitive regulation and perceived vocabulary retention during ChatGPT-assisted learning rather than direct behavioural assessments of working memory capacity, attentional control, or objective vocabulary retention performance. In addition, because the study employed a cross-sectional correlational design in which all variables were measured at a single time point, the structural model tests relationships consistent with the proposed theoretical framework, but does not establish temporal precedence or causal mediation among the constructs. Prior research has shown that self-report indicators of cognitive ability often display modest associations with objective cognitive tests (Friedman & Robbins, 2021; Li, 2023), suggesting that they represent perceived regulatory behaviour rather than underlying cognitive capacity. Future studies could therefore combine self-report measures with behavioural or performance-based cognitive assessments in order to provide a more comprehensive representation of the cognitive processes involved in AI-assisted vocabulary learning. Another limitation concerns the operationalisation of frequency of ChatGPT use as a single conditioning variable, which did not distinguish between qualitatively different engagement patterns, such as brief repetitive exchanges versus extended interactional sequences or input-oriented versus output-oriented use; subsequent research should disaggregate frequency using interaction logs or learning analytics to identify which forms of repeated engagement most strongly condition cognitive regulation. A third limitation relates to the exclusive focus on vocabulary retention as the learning outcome, which, although theoretically justified, does not capture whether the same cognitive pathways apply to other aspects of language development such as lexical diversity in production, fluency, or form–meaning integration during speaking (Kormos, 2023; Teng & Zhang, 2023); future work should test the stability of the proposed model across multiple receptive and productive language outcomes. A further limitation arises from the interactional properties of ChatGPT itself: AI-mediated communication lacks the social contingency and pragmatic variability characteristic of human–human interaction, which may, in turn, shape attentional regulation in different ways. Future studies should compare AI-mediated and human-mediated interactions to determine whether the observed cognitive pathways are specific to conversational AI contexts. Finally, the absence of systematic task manipulation in ChatGPT interactions limits conclusions about how varying interactional demands influence cognitive regulation; future research should manipulate task complexity, lexical load, or output constraints to examine how changes in conversational demands alter the deployment of working memory and attentional control during AI-assisted foreign language learning.

## 8. Conclusions

This study set out to clarify how cognitive regulation structures vocabulary retention during ChatGPT-assisted foreign language learning. The results show that learning outcomes in conversational AI settings are shaped by the coordinated functioning of working memory and attention control, with attention control determining whether conversational input is stabilised as retained lexical knowledge. Working memory supported retention directly and through its role in sustaining attentional regulation during interaction, indicating that conversational engagement places sustained demands on learners’ regulatory capacity. Frequency of ChatGPT use conditioned these relationships by shaping the interactional context in which cognitive resources were applied, strengthening regulatory efficiency across repeated engagement without functioning as an independent source of learning gains. These findings frame ChatGPT-assisted vocabulary learning as a cognitively regulated activity rather than an exposure-driven process, advancing a process-oriented account of AI-mediated language learning that situates individual differences in cognitive control at the centre of vocabulary development.

## Figures and Tables

**Table 1:** Demographic profiles.

Variable	Category	Frequency	Percent
Gender	Male	538	53.7
	Female	464	46.3
Age group	18–22 years	412	41.1
	23–26 years	361	36.0
	27–30 years	152	15.2
	31 years and above	77	7.7
University level	First year	226	22.6
	Second year	271	27.0
	Third year	289	28.8
	Fourth year and above	216	21.6
Frequency of ChatGPT use	Rarely	144	14.4
	Sometimes	318	31.7
	Often	356	35.5
	Very often	184	18.4
Self-rated English proficiency	Beginner	146	14.6
	Intermediate	418	41.7
	Upper-intermediate	308	30.7
	Advanced	130	13.0

**Table 2:** KMO and BTS test.

Scale	KMO	Bartlett’s χ^2^	df	*p*
Working Memory	0.82	1214.47	120	<.001
Attention Control	0.84	1687.92	120	<.001
Vocabulary Retention	0.81	1948.63	120	<.001

**Table 3:** Measurement fit indices.

Fit Index	Working Memory	Attention Control	Vocabulary Retention
χ^2^	312.84	289.17	301.56
df	164	164	164
χ^2^/df	1.91	1.76	1.84
CFI	0.957	0.962	0.955
TLI	0.948	0.954	0.946
RMSEA	0.042	0.039	0.041
SRMR	0.041	0.038	0.043

**Table 4:** Convergence and discriminant validity.

Sub-Scale	α	CR	AVE	1	2	3	4
Working Memory Scale							
1 = Phonological Storage	0.77	0.76	0.52	0.72			
2 = Processing Efficiency	0.83	0.83	0.55	0.54	0.74		
3 = Updating Ability	0.85	0.85	0.59	0.50	0.58	0.77	
4 = Inhibition Control	0.75	0.75	0.50	0.46	0.52	0.56	0.71
Attention Control Scale							
1 = Sustained Attention	0.85	0.86	0.61	0.78			
2 = Selective Attention	0.81	0.81	0.59	0.61	0.77		
3 = Attention Switching	0.82	0.82	0.60	0.56	0.59	0.77	
4 = Inhibitory Control	0.84	0.84	0.57	0.58	0.55	0.57	0.75
Vocabulary Retention Scale							
1 = Immediate Recall	0.80	0.81	0.58	0.76			
2 = Delayed Retention	0.81	0.81	0.58	0.63	0.76		
3 = Semantic Integration	0.79	0.79	0.55	0.46	0.41	0.74	
4 = Productive Use	0.78	0.77	0.53	0.54	0.55	0.59	0.72

**Table 5:** Common latent factor.

Scale	Item	Loading (Before CLF)	Loading (After CLF)	Difference
Working Memory	PS1	0.72	0.70	0.02
	PS2	0.75	0.73	0.02
	PS4	0.69	0.67	0.02
	PE1	0.74	0.72	0.02
	PE2	0.77	0.75	0.02
	PE3	0.71	0.69	0.02
	PE4	0.73	0.71	0.02
	UA1	0.76	0.74	0.02
	UA2	0.79	0.77	0.02
	UA3	0.74	0.72	0.02
	UA4	0.77	0.75	0.02
	IC1	0.71	0.69	0.02
	IC2	0.69	0.67	0.02
	IC3	0.73	0.71	0.02
Attention Control	SA1	0.78	0.76	0.02
	SA2	0.75	0.73	0.02
	SA3	0.81	0.78	0.03
	SA4	0.73	0.71	0.02
	SAt1	0.72	0.70	0.02
	SAt2	0.76	0.74	0.02
	SAt3	0.76	0.72	0.02
	SAt4	0.76	0.75	0.01
	AS2	0.74	0.72	0.02
	AS3	0.79	0.77	0.02
	AS4	0.73	0.71	0.02
	ICn1	0.75	0.73	0.02
	ICn2	0.78	0.76	0.02
	ICn3	0.72	0.70	0.02
	ICn4	0.70	0.68	0.02
Vocabulary Retention	IR2	0.81	0.79	0.02
	IR3	0.75	0.73	0.02
	IR4	0.73	0.71	0.02
	DR1	0.76	0.74	0.02
	DR2	0.79	0.77	0.02
	DR3	0.74	0.72	0.02
	SI1	0.77	0.75	0.02
	SI3	0.74	0.72	0.02
	SI4	0.72	0.70	0.02
	PU1	0.76	0.74	0.02
	PU2	0.73	0.71	0.02
	PU4	0.70	0.68	0.02

**Table 6:** Multicollinearity diagnostics for structural model predictors.

Endogenous Equation	Predictor	VIF	Tolerance
Attention Control	Working Memory	1.83	0.55
	Frequency of ChatGPT use	1.26	0.79
	Working Memory × Frequency	1.98	0.50
	English proficiency	1.34	0.75
Vocabulary Retention	Working Memory	1.92	0.52
	Attention Control	2.14	0.47
	Frequency of ChatGPT use	1.31	0.76
	Attention Control × Frequency	2.22	0.45
	English proficiency	1.38	0.72
	University level	1.17	0.85

**Table 7:** Direct, mediation, and moderation effect model.

Path/Hypothesis	Std. Est. (β)	SE	95% CI	*p*
Direct effects				
H1: Working Memory → Attention Control	0.54	0.04	[0.46, 0.62]	<.001
H2: Working Memory → Vocabulary Retention	0.18	0.05	[0.08, 0.28]	<.001
H3: Attention Control → Vocabulary Retention	0.46	0.04	[0.38, 0.54]	<.001
Indirect (mediated) effects				
H4: Working Memory → Attention Control → Vocabulary Retention	0.25	0.03	[0.19, 0.31]	<.001
Moderation effects				
H5: Working Memory × ChatGPT Use → Attention Control	0.14	0.04	[0.06, 0.22]	.001
H6: Attention Control × ChatGPT Use → Vocabulary Retention	0.17	0.05	[0.07, 0.27]	.002
Covariates				
English Proficiency → Working Memory	0.31	0.04	[0.23, 0.39]	<.001
English Proficiency → Attention Control	0.28	0.04	[0.20, 0.36]	<.001
English Proficiency → Vocabulary Retention	0.22	0.05	[0.12, 0.32]	<.001
University Level → Vocabulary Retention	0.12	0.04	[0.04, 0.20]	.004

## Data Availability

The data presented in this study are available on request from the corresponding author due to ethical and privacy considerations.

## Supplementary Materials

The following supporting information can be downloaded at: https://www.mdpi.com/article/10.3390/jintelligence14040062/s1, Figure S1: Scree plot for the three scales; Table S1: Descriptive statistics and zero-order correlation of scale constructs; Table S2: Total variance explained for the Working Memory scale; Table S3: Total variance explained for the Attention Control scale; Table S4: Total variance explained for the Vocabulary Retention scale; Table S5: Rotated Component Matrix for Working Memory scale; Table S6: Rotated Component Matrix for Attention Control scale; Table S7: Rotated Component Matrix for Vocabulary Retention scale.
