# Supplementary material for: Working Memory, Attention Control, and Vocabulary Retention in AI (ChatGPT)-Assisted Foreign Language Learning: A Structural Cognitive Modelling Approach
Source: J Intell. 2026 Apr 8;14(4):62. doi: 10.3390/jintelligence14040062 (PMC13117997; doi:10.3390/jintelligence14040062)
Supplement: Supplementary file 1 [file jintelligence-14-00062-s001.zip › jintelligence-4171661-supplementary.pdf]

Supplementary Materials

This file provides additional materials supporting the analysis, including the survey instrument, descriptive statistics, and detailed exploratory factor analysis (EFA) results.

Response Scale: 1 = Strongly Disagree 2 = Disagree 3 = Neutral 4 = Agree 5 = Strongly Agree

**Demographic Information**			
Gender	☐ Male ☐ Female		
Age	☐ 18–22 ☐ 23–26 ☐ 27–30 ☐ 31 +		
University level	☐ First year ☐ Second year ☐ third year ☐ fourth year and above		
Frequency of ChatGPT Use	☐ Rarely ☐ Sometimes ☐ Often ☐ Very Often		
Self-Rated English Proficiency	☐ Beginner ☐ Intermediate ☐ Upper-Intermediate ☐ Advanced		

**Section / Dimension**	**Code**	**Item Statement**	**Response Scale (1–5)**
**Construct 1: Working Memory**			
**Phonological Storage**	1.	I can remember several unfamiliar words without writing them down.	1 2 3 4 5
	2.	When ChatGPT suggests new vocabulary, I can keep those words in mind while finishing the task.	1 2 3 4 5
	3.	I can mentally repeat new words from ChatGPT to help me remember them.	1 2 3 4 5
	4.	I can hold new phrases in memory while planning my response.	1 2 3 4 5
**Processing Efficiency**	5.	I can process several pieces of linguistic information at the same time.	1 2 3 4 5
	6.	I can mentally organise ChatGPT's ideas before I start writing.	1 2 3 4 5
	7.	I can remember grammar rules while using them during ChatGPT tasks.	1 2 3 4 5
	8.	I can mentally integrate ChatGPT's linguistic feedback while formulating my own sentences.	1 2 3 4 5
**Updating Ability**	9.	I can easily update word meanings when ChatGPT provides clarifications.	1 2 3 4 5
	10.	I can revise earlier vocabulary choices after new input from ChatGPT.	1 2 3 4 5

	11.	I can replace less accurate words with better ones I recently learned.	1 2 3 4 5
	12.	I can correct earlier vocabulary mistakes after feedback.	1 2 3 4 5
**Inhibition Control**	13.	I can ignore irrelevant ChatGPT suggestions when learning new words.	1 2 3 4 5
	14.	I can block distractions from unrelated ChatGPT topics.	1 2 3 4 5
	15.	I can focus on target vocabulary even when many words appear together.	1 2 3 4 5
	16.	I avoid confusion between similar-sounding foreign words.	1 2 3 4 5
**Construct 2: Attention Control**			
**Sustained Attention**	17.	I can stay focused on ChatGPT-assisted tasks for an extended period.	1 2 3 4 5
	18.	I rarely lose concentration while using ChatGPT for vocabulary learning.	1 2 3 4 5
	19.	I can stay mentally engaged throughout a ChatGPT session.	1 2 3 4 5
	20.	I can continue working on ChatGPT exercises without being distracted by other tasks.	1 2 3 4 5
**Selective Attention**	21.	I consciously choose which vocabulary items to study first.	1 2 3 4 5
	22.	I can quickly identify which ChatGPT suggestions are worth learning.	1 2 3 4 5
	23.	I pay attention only to the examples that support my learning goal.	1 2 3 4 5
	24.	I can separate important vocabulary explanations from less relevant ones.	1 2 3 4 5
**Attention Switching**	25.	I can shift attention smoothly between reading ChatGPT output and writing my own sentences.	1 2 3 4 5
	26.	I adapt easily when ChatGPT introduces new words or topics.	1 2 3 4 5
	27.	I can manage my attention while moving between comprehension and production tasks.	1 2 3 4 5
	28.	I can handle multiple ChatGPT prompts without losing focus.	1 2 3 4 5
**Inhibitory Control**	29.	I can resist automatically accepting ChatGPT's vocabulary suggestions.	1 2 3 4 5
	30.	I can stop myself from copying ChatGPT's words without reflection.	1 2 3 4 5

	31.	I can keep my attention on one linguistic goal despite several prompts.	1 2 3 4 5
	32.	I can suppress unrelated thoughts while concentrating on vocabulary.	1 2 3 4 5
**Construct 3: Vocabulary Retention**			
**Immediate Recall**	33.	I can remember new words immediately after learning them with ChatGPT.	1 2 3 4 5
	34.	I can recall new vocabulary just after completing a ChatGPT activity.	1 2 3 4 5
	35.	I can immediately recall newly learned words from ChatGPT in short sentences.	1 2 3 4 5
	36.	I recognise words I just learned when they reappear in ChatGPT dialogue.	1 2 3 4 5
**Delayed Retention**	37.	I can recall ChatGPT-taught words several days later.	1 2 3 4 5
	38.	I can recognise previously learned words after one or two weeks.	1 2 3 4 5
	39.	I can retain new vocabulary over time through ChatGPT interaction.	1 2 3 4 5
	40.	I can recall most of the words I practised in past ChatGPT sessions.	1 2 3 4 5
**Semantic Integration**	41.	I can understand new words in various contexts after learning them.	1 2 3 4 5
	42.	I can link new words to related ideas or synonyms from ChatGPT.	1 2 3 4 5
	43.	I can interpret the differences in meaning among ChatGPT's vocabulary examples.	1 2 3 4 5
	44.	I can relate new vocabulary to words I already know.	1 2 3 4 5
**Productive Use**	45.	I can use newly learned words correctly in my writing.	1 2 3 4 5
	46.	I can apply ChatGPT-taught vocabulary in new sentences.	1 2 3 4 5
	47.	I can use learned words appropriately in speaking tasks.	1 2 3 4 5
	48.	I can select the right words when writing independently after learning them.	1 2 3 4 5

Table S1. Descriptive statistics and zero-order correlation of scale constructs

Variable	M	SD	1	2	3	4
1. Working Memory	3.68	0.54	1.0			
2. Attention Control	3.74	0.57	.61***	1.0		
3. Vocabulary Retention	3.81	0.52	.42***	.58***	1.0	
4. ChatGPT Use	3.58	0.91	.29***	.34***	.31***	1.0

***$p < 0.01$

Table S2. *Total variance explained for the Working Memory scale.*

**Component**	**Eigenvalue**	**% of Variance**	**Cumulative %**
**1**	5.20	32.50	32.50
**2**	2.40	15.00	47.50
**3**	1.80	11.25	58.75
**4**	1.63	10.19	68.94
**5**	0.75	4.69	73.63
**6**	0.67	4.19	77.81
**7**	0.60	3.75	81.56
**8**	0.54	3.38	84.94
**9**	0.49	3.06	88.00
**10**	0.44	2.75	90.75
**11**	0.35	2.19	92.94
**12**	0.31	1.94	94.88
**13**	0.27	1.69	96.56
**14**	0.23	1.44	98.00
**15**	0.19	1.19	99.19
**16**	0.13	0.81	100.00

Table S3. *Total variance explained for the Attention Control scale.*

**Component**	**Eigenvalue**	**% of Variance**	**Cumulative %**
**1**	5.79	36.19	36.19
**2**	2.41	15.06	51.25
**3**	1.66	10.38	61.63
**4**	1.25	7.81	69.44
**5**	0.75	4.69	74.13
**6**	0.65	4.06	78.19
**7**	0.56	3.50	81.69
**8**	0.48	3.00	84.69
**9**	0.42	2.63	87.31
**10**	0.40	2.50	89.81
**11**	0.35	2.19	92.00
**12**	0.30	1.88	93.88
**13**	0.28	1.75	95.63
**14**	0.26	1.62	97.25
**15**	0.24	1.50	98.75
**16**	0.20	1.25	100.00

Table S4. *Total variance explained for the Vocabulary Retention Scale.*

Component	Eigenvalue	% of Variance	Cumulative %
**1**	6.14	38.38	38.38
**2**	2.31	14.44	52.82
**3**	1.57	9.81	62.63
**4**	1.19	7.44	70.07
**5**	0.64	4.00	74.07
**6**	0.58	3.62	77.69
**7**	0.52	3.25	80.94
**8**	0.46	2.88	83.82
**9**	0.42	2.62	86.44
**10**	0.39	2.44	88.88
**11**	0.36	2.25	91.13
**12**	0.33	2.06	93.19
**13**	0.31	1.94	95.13
**14**	0.28	1.75	96.88
**15**	0.25	1.56	98.44
**16**	0.25	1.56	100.00

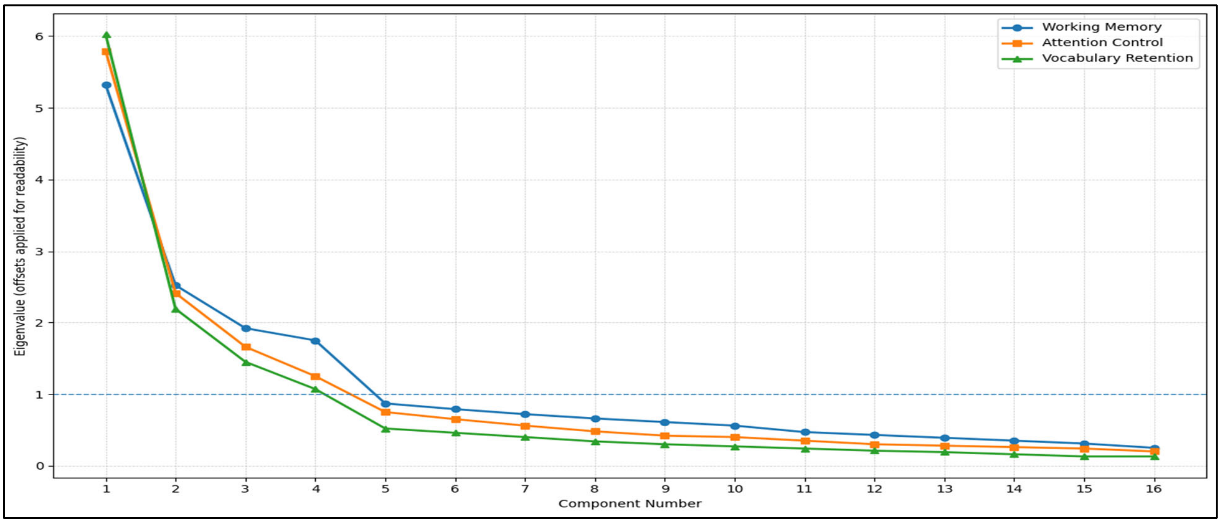

Figure S1. *Scree plot for the three scales.*

Table S5. *Rotated Component Matrix for Working Memory Scale.*

Item	1	2	3	4
**PS1**	**.72**	.18	.11	.09
**PS2**	**.75**	.21	.14	.08
**PS3***	.28	.19	.10	.07
**PS4**	**.69**	.23	.16	.12
**PE1**	.20	**.74**	.18	.14
**PE2**	.22	**.77**	.21	.13
**PE3**	.19	**.71**	.25	.17
**PE4**	.24	**.73**	.22	.15
**UA1**	.15	.20	**.76**	.18
**UA2**	.17	.22	**.79**	.19

**UA3**	.14	.18	**.74**	.23
**UA4**	.16	.21	**.77**	.20
**IC1**	.12	.19	.24	**.71**
**IC2**	.10	.17	.21	**.69**
**IC3**	.18	.22	.26	**.73**
**IC4***	.28	.21	.19	.27

Table S6. *Rotated Component Matrix for Attention Control Scale.*

Item	1	2	3	4
**SA1**	**.78**	.16	.12	.09
**SA2**	**.75**	.18	.14	.11
**SA3**	**.81**	.15	.10	.08
**SA4**	**.73**	.20	.17	.13
**SAt1**	.19	**.72**	.21	.14
**SAt2**	.17	**.76**	.18	.12
**SAt3**	.21	**.74**	.20	.16
**SAt4**	.18	**.61**	.23	.15
**AS1***	.14	.19	**.27**	.17
**AS2**	.16	.22	**.74**	.19
**AS3**	.20	.18	**.79**	.15
**AS4**	.23	.21	**.73**	.18
**ICn1**	.12	.17	.19	**.75**
**ICn2**	.10	.15	.22	**.78**
**ICn3**	.14	.20	.24	**.72**
**ICn4**	.16	.18	.21	**.70**

Table S7. *Rotated Component Matrix for Vocabulary Retention Scale.*

Item	1	2	3	4
**IR1***	.18	.14	.11	.09
**IR2**	**.81**	.16	.10	.08
**IR3**	**.75**	.18	.13	.12
**IR4**	**.73**	.19	.15	.14
**DR1**	.17	**.76**	.14	.11
**DR2**	.15	**.79**	.16	.12
**DR3**	.19	**.74**	.18	.15
**DR4***	.21	.11	.20	.17
**SI1**	.14	.18	**.77**	.16
**SI2***	.16	.20	.29	.19
**SI3**	.18	.22	**.74**	.21
**SI4**	.20	.19	**.72**	.23
**PU1**	.22	.24	.27	**.76**
**PU2**	.19	.21	.29	**.73**
**PU3***	.26	.28	.29	.27
**PU4**	.24	.26	.28	**.79**
